# Supplementary material for: Strain-induced room-temperature ferroelectricity in SrTiO3 membranes
Source: Nat Commun. 2020 Jun 19;11:3141. doi: 10.1038/s41467-020-16912-3 (PMC7305178; doi:10.1038/s41467-020-16912-3)
Supplement: Supplementary file 1 — Supplementary Information [file 41467_2020_16912_MOESM1_ESM.pdf]

# Supplementary Information

# Strain-Induced Room-Temperature Ferroelectricity in $SrTiO_3$ Membranes

**Xu et al.**

**Supplementary Figure 1**

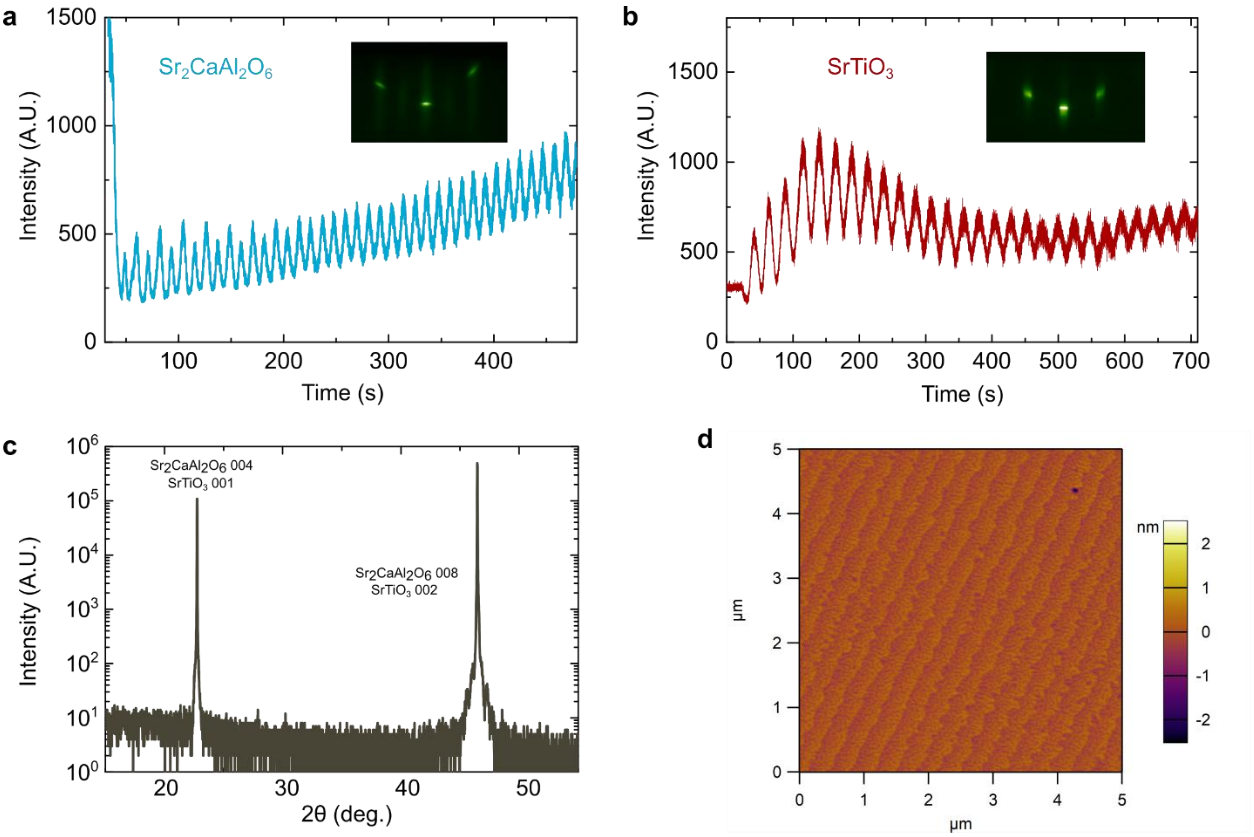


**Supplementary Figure 1 | Growth and structural characterization of $SrTiO_3$ / $Sr_2CalAl_2O_6$ / $SrTiO_3$ heterostructure.** Reflection high-energy electron diffraction (RHEED) oscillations and patterns of **a**, $Sr_2CaAl_2O_6$ and **b**, $SrTiO_3$, which indicate typical layer-by-layer 2D growth mode. **c**, θ - 2θ X-ray diffraction scans showing a single diffraction peak with all layer peaks overlapping together due to the small lattice mismatch between the different film layers. **d**, Atomic force microscopy characterization of as-grown $SrTiO_3$ films showing an atomically smooth surface with unit cell step terraces.

**Supplementary Figure 2**

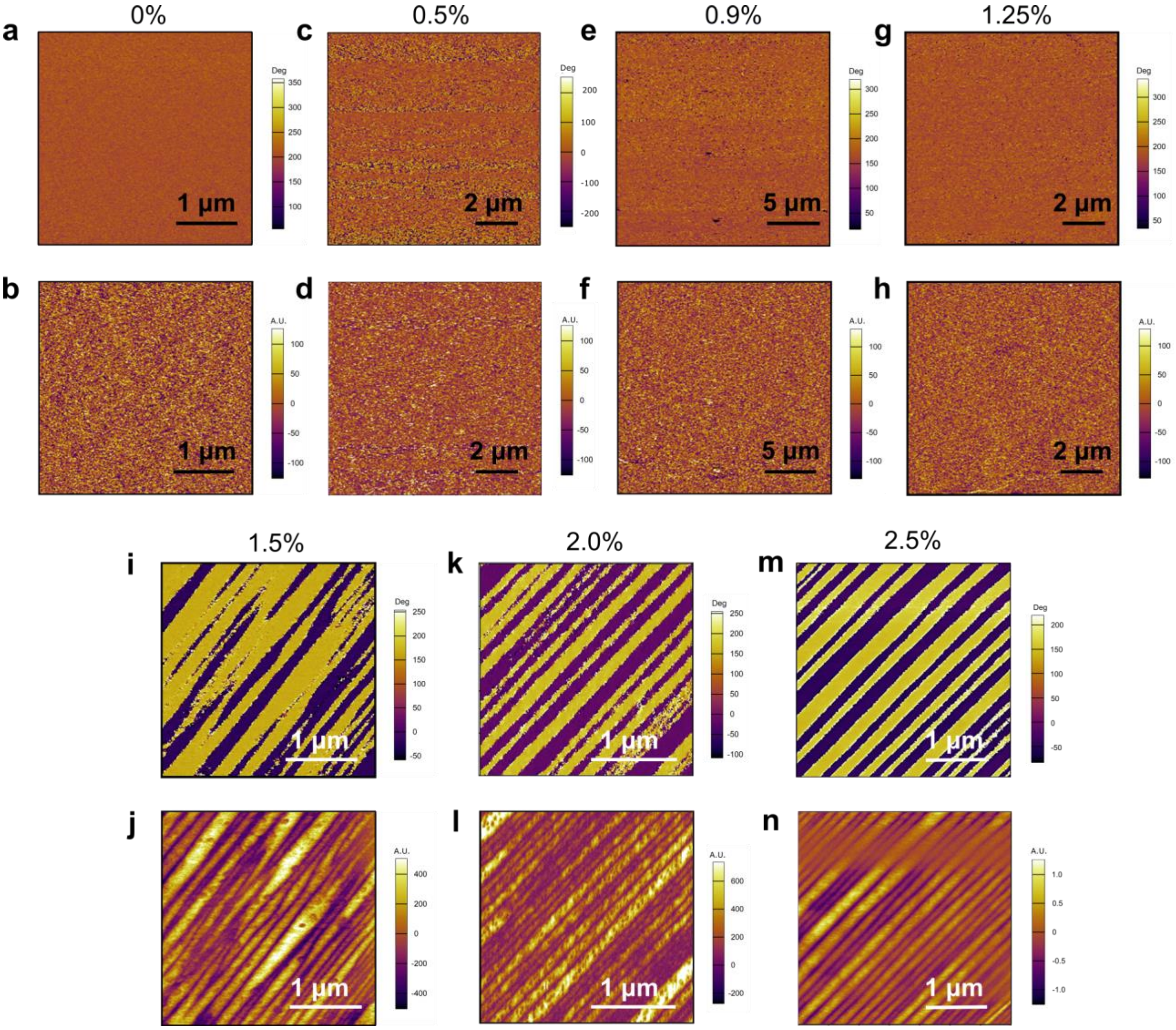


**Supplementary Figure 2 | Room-temperature piezoresponse force microscopy (PFM) characterization of strained $SrTiO_3$ membranes.** Lateral PFM phase and amplitude of membranes uniaxially strained at **a**, **b**, 0%; **c**, **d**, 0.5%; **e**, **f**, 0.9%; **g**, **h**, 1.25%; **i**, **j**, 1.5%; **k**, **l**, 2.0%; **m**, **n**, 2.5%, respectively, wherein room-temperature ferroelectricity is observed to emerge at 1.5% strain, which is characterized by the 180° ferroelectric polydomain patterns.

**Supplementary Figure 3**

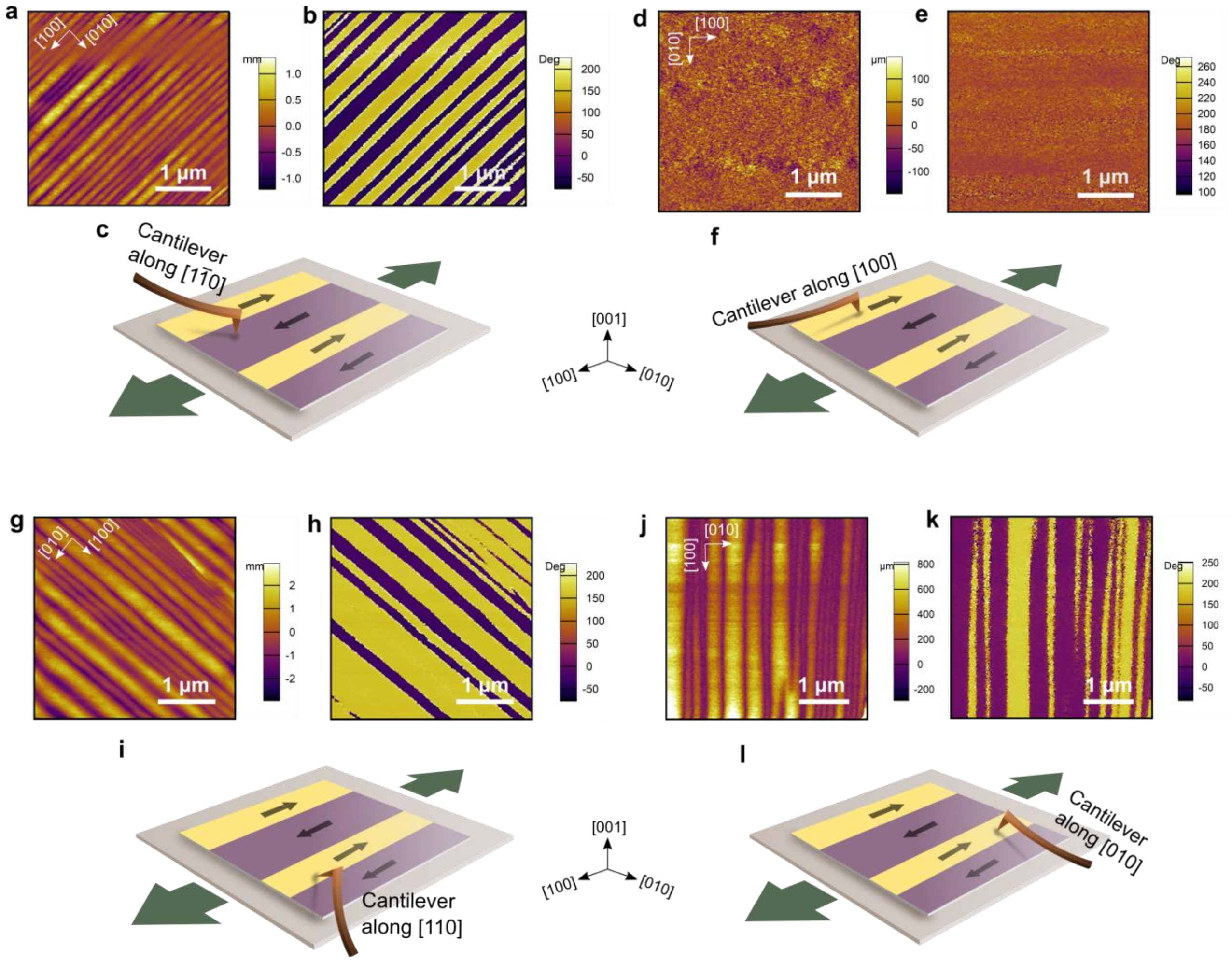


**Supplementary Figure 3 | Piezoresponse force microscopy (PFM) characterization of domain structures in 2.5% strained $SrTiO_3$ membranes.** Lateral PFM amplitude and phase measured by aligning the PFM cantilever along the **a**, **b**, [1$\bar{1}$0], **d**, **e**, [100], **g**, **h**, [110], and **j**, **k**, [010] directions, respectively. Whereas evident ferroelectric stripe domain patterns are observed when scanning along the [1$\bar{1}$0], [110], and [010] directions, the piezoresponse vanishes when scanning along [100], indicating the in-plane polarization of the ferroelectric domains is aligned along [100]/[$\bar{1}$00], with the adjacent domains polarized at a 180° difference. The measurement process is illustrated in **c**, **f**, **i**, and **l**. These results clearly show that the polarization of these domain features

align along specific crystallographic directions, which evidences the presence of ferroelectricity, whereas other mechanisms of induced ferroelectric-like PFM response are typically invariant with respect to the cantilever scan orientation.

**Supplementary Figure 4**

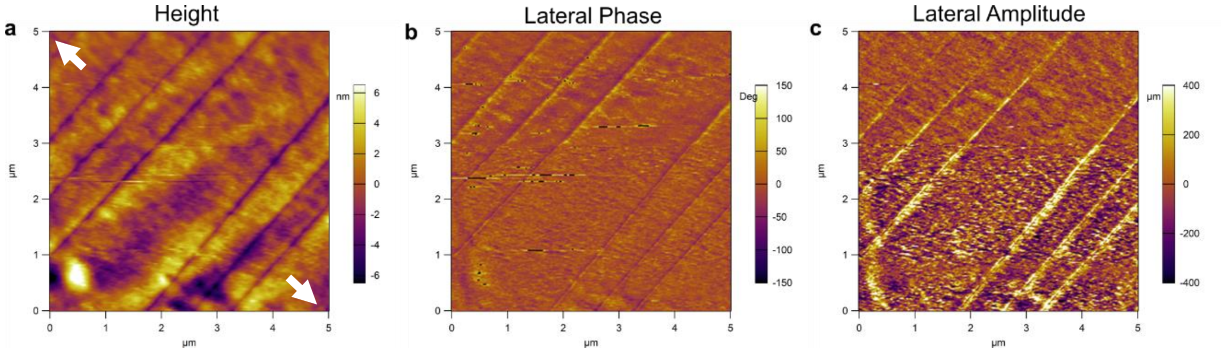


**Supplementary Figure 4 | Piezoresponse Force Microscopy (PFM) characterization of strained $SrTiO_3$ membranes beyond the threshold for crack formation**. **a**, PFM height image shows cracks perpendicular to the uniaxial strain direction. White arrows illustrate the uniaxial strain direction along [100]. **b**, Lateral phase and **c**, amplitude images show the absence of 180º degree domains in cracked $SrTiO_3$ membranes.

**Supplementary Figure 5**

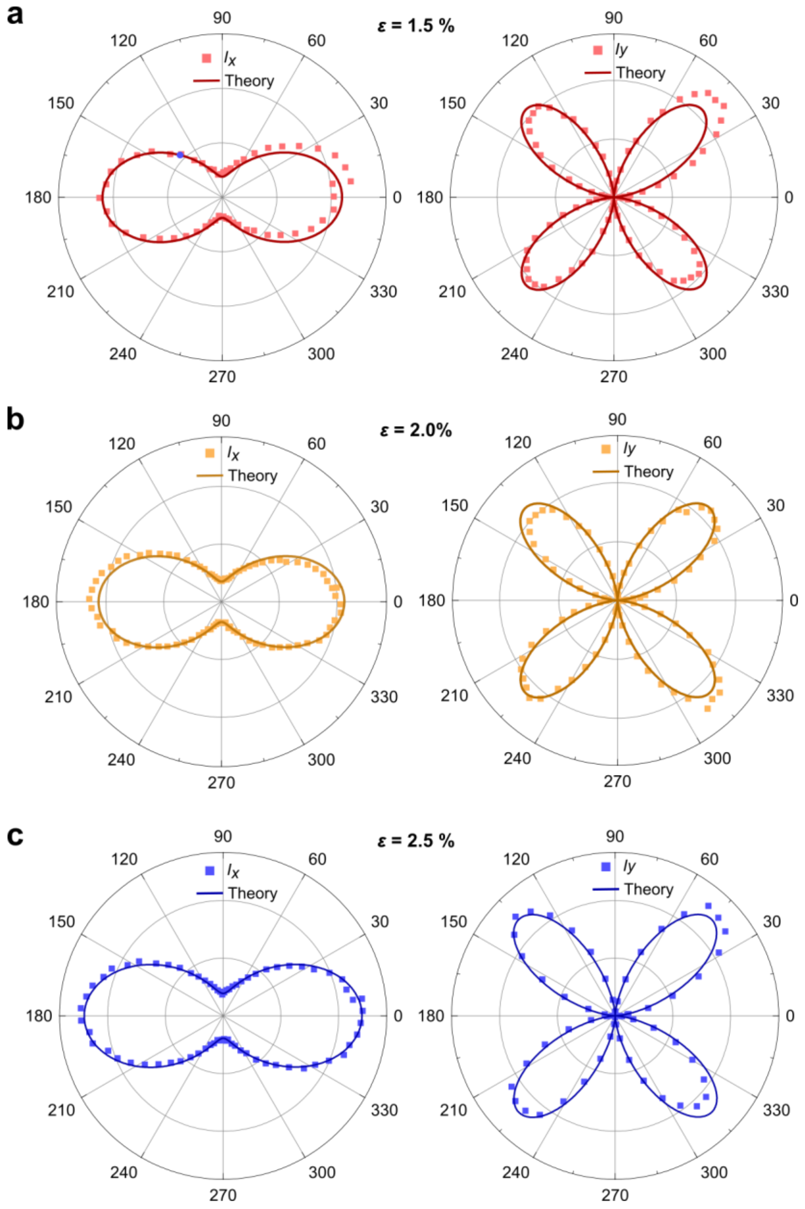


**Supplementary Figure 5 | Optical second harmonic generation (SHG) measurements of $SrTiO_3$ membranes.** SHG polar plots measured at room temperature as a function of incident beam polarization in membranes strained at **a**, 1.5%, **b**, 2.0%, **c**, 2.5%.

**Supplementary Figure 6**

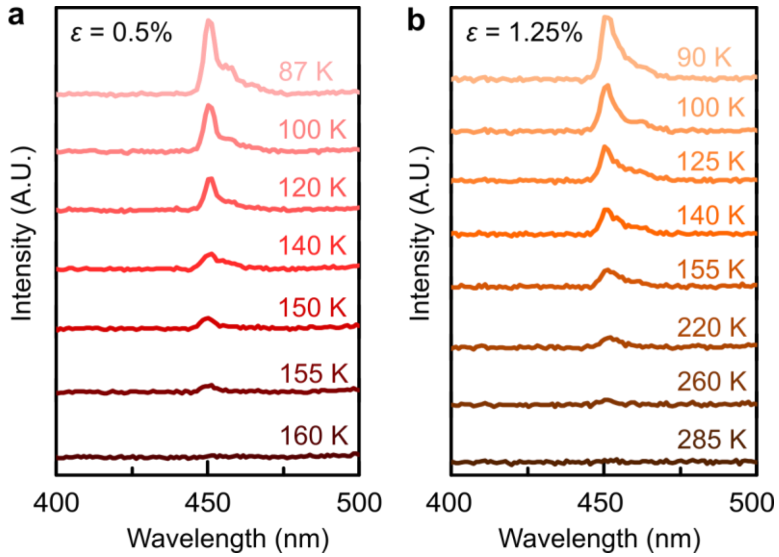


**Supplementary Figure 6 | Temperature-dependent second harmonic generation (SHG) measurements of strained $SrTiO_3$ membranes.** SHG measurements performed on $SrTiO_3$ membranes as a function of temperature. The membranes are uniaxially strained at **a**, 0.5% and **b**, 1.25%.

**Supplementary Figure 7**

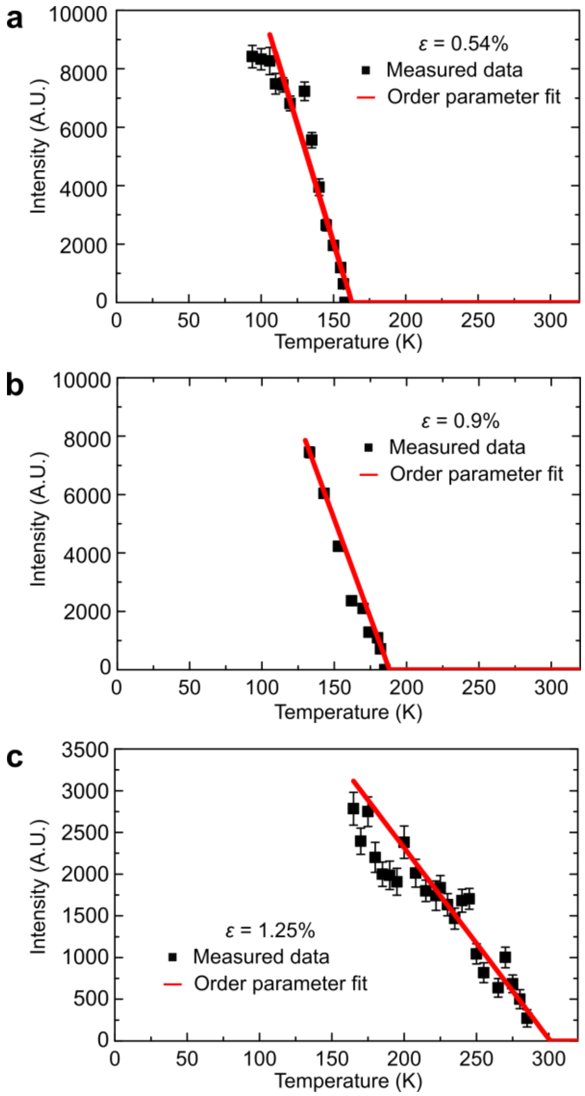


**Supplementary Figure 7 | Extracting $T_c$ from SHG results for each strain state.** Here, since the SHG intensity $\propto P_1^2$ while $P_1 \propto T^{1/2}$ (within the Ginsburg-Landau-Devonshire model), we can derive that the SHG intensity $\propto T$. Plotting the integrated SHG peak intensity as a function of temperature for membranes uniaxially strained at **a**, 0.54%, **b**, 0.9%, and **c**, 1.25%, the $T_c$ can be extracted for each strain state with this linear intensity-temperature relationship. Error bars represent the standard deviation.

**Supplementary Figure 8**

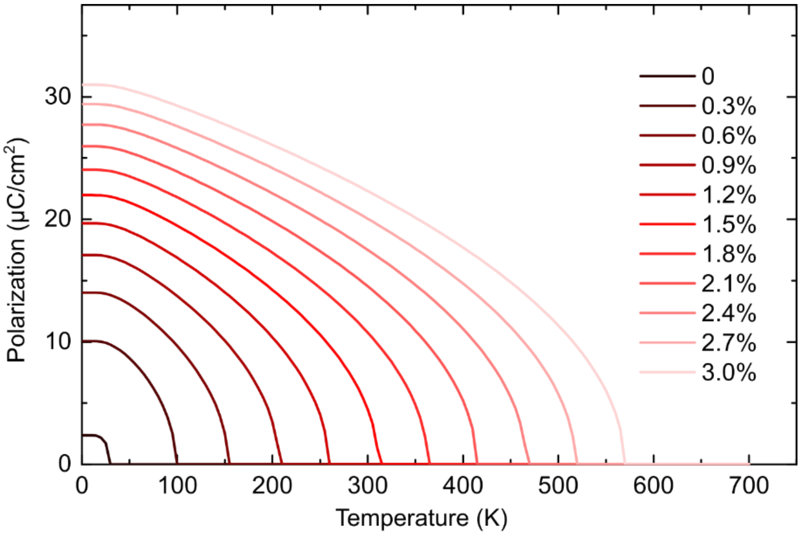


**Supplementary Figure 8 | The calculated polarization evolution with temperature at different uniaxial strain states.** Temperature-dependent polarization evolution as a function of applied uniaxial strain along [100] is calculated using the Ginsburg-Landau-Devonshire model. The relation between the transition temperature $T_c$ and strain can be further extracted at $P = 0$.

**Supplementary Figure 9**

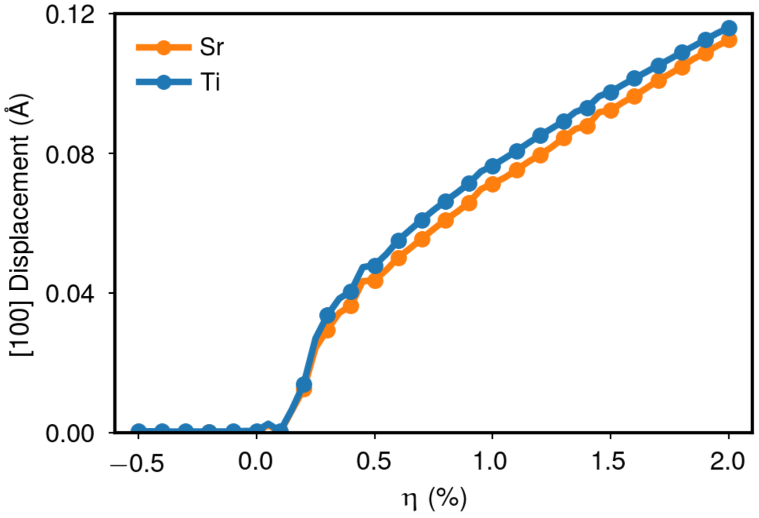


**Supplementary Figure 9 | The local displacements of Sr and Ti atoms calculated by DFT**. Both the Sr and Ti atoms in $SrTiO_3$ displace along the [100] direction in response to the uniaxial strain along [100].

**Supplementary Figure 10**

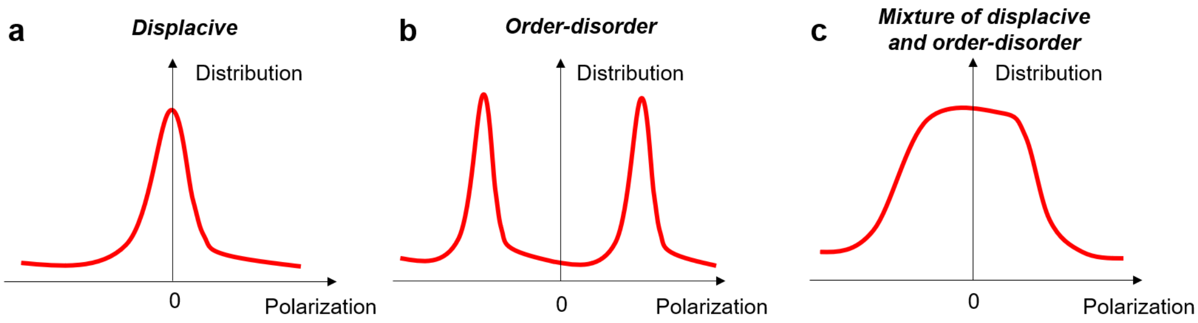


**Supplementary Figure 10 | Schematics of the characteristic polarization distribution near the phase transition**. The polarization distribution in the paraelectric phase for **a**, displacive, **b**, order-disorder, and **c**, a mixture of the two. In the ferroelectric phase of strained $SrTiO_3$, the displacive character dominates at lower temperature, as the distribution of local polarization has a single peak and the peak position shifts toward lower polarization values with increasing temperature. When the temperature rises close to $T_c$, the order-disorder character dominates, shown as a double-peak in the distribution curve. However, an ideal order-disorder character will have the two peaks well separated in the high temperature phase. Therefore, even at a temperature close to $T_c$, the displacive character still plays a role, indicating a mixture of displacive and order-disorder transition characteristics in $SrTiO_3$.

**Supplementary Table 1. DFT calculated structural evolution with uniaxial strain**

| Strain (%) | *a* (Å) | *b* (Å) | *c* (Å) | $P_x$ (C m$^{-2}$) | $P_y$ (C m$^{-2}$) | $P_z$ (C m$^{-2}$) |
|---|---|---|---|---|---|---|
| -0.5 | 3.8377 | 3.857 | 3.8617 | 0.001 | 0 | 0 |
| -0.45 | 3.8396 | 3.857 | 3.8617 | 0.001 | 0 | 0 |
| -0.4 | 3.8416 | 3.857 | 3.8606 | 0.001 | 0 | 0 |
| -0.35 | 3.8435 | 3.857 | 3.86 | 0.001 | 0 | 0 |
| -0.3 | 3.8454 | 3.857 | 3.8595 | 0.001 | 0 | 0 |
| -0.25 | 3.8474 | 3.857 | 3.859 | 0.001 | 0 | 0 |
| -0.2 | 3.8493 | 3.857 | 3.8586 | 0.001 | 0 | 0 |
| -0.15 | 3.8512 | 3.857 | 3.8578 | 0.001 | 0 | 0 |
| -0.1 | 3.8531 | 3.857 | 3.8573 | 0.001 | 0 | 0 |
| -0.05 | 3.8551 | 3.857 | 3.8548 | 0.001 | 0 | 0 |
| 0 | 3.857 | 3.857 | 3.8548 | 0.001 | 0 | 0 |
| 0.05 | 3.8589 | 3.857 | 3.8554 | -0.007 | 0 | 0 |
| 0.1 | 3.8609 | 3.857 | 3.855 | 0.001 | 0 | 0 |
| 0.15 | 3.8628 | 3.857 | 3.8543 | 0.018 | 0 | 0 |
| 0.2 | 3.8647 | 3.857 | 3.8539 | 0.037 | 0 | 0 |
| 0.25 | 3.8666 | 3.857 | 3.8534 | 0.072 | 0.001 | 0 |
| 0.3 | 3.8686 | 3.857 | 3.8529 | 0.09 | 0.001 | 0 |
| 0.35 | 3.8705 | 3.857 | 3.8525 | 0.103 | 0.001 | 0 |
| 0.4 | 3.8724 | 3.857 | 3.8516 | 0.109 | 0.001 | 0 |
| 0.45 | 3.8744 | 3.857 | 3.8515 | 0.128 | 0.001 | 0 |
| 0.5 | 3.8763 | 3.857 | 3.8508 | 0.129 | 0.001 | 0 |
| 0.55 | 3.8782 | 3.857 | 3.8501 | 0.137 | 0.002 | 0 |
| 0.6 | 3.8801 | 3.857 | 3.8496 | 0.148 | 0.002 | 0 |
| 0.65 | 3.8821 | 3.857 | 3.8493 | 0.156 | 0.002 | 0 |
| 0.7 | 3.884 | 3.857 | 3.8488 | 0.164 | 0.002 | 0 |
| 0.75 | 3.8859 | 3.857 | 3.8482 | 0.171 | 0.002 | 0 |
| 0.8 | 3.8878 | 3.857 | 3.8472 | 0.178 | 0.002 | 0 |
| 0.85 | 3.8898 | 3.857 | 3.8466 | 0.185 | 0.002 | 0 |
| 0.9 | 3.8917 | 3.857 | 3.8466 | 0.192 | 0.003 | 0 |
| 0.95 | 3.8936 | 3.857 | 3.8455 | 0.202 | 0.003 | 0 |
| 1 | 3.8956 | 3.857 | 3.8454 | 0.206 | 0.003 | 0 |
| 1.05 | 3.8975 | 3.857 | 3.8448 | 0.211 | -0.007 | 0.001 |
| 1.1 | 3.8994 | 3.857 | 3.8443 | 0.217 | -0.007 | 0.001 |
| 1.15 | 3.9013 | 3.857 | 3.8417 | 0.224 | -0.007 | 0.001 |
| 1.2 | 3.9033 | 3.857 | 3.8425 | 0.229 | -0.007 | 0.001 |
| 1.25 | 3.9052 | 3.857 | 3.8405 | 0.235 | -0.007 | 0.001 |
| 1.3 | 3.9071 | 3.857 | 3.8412 | 0.241 | -0.007 | 0.001 |
| 1.35 | 3.9091 | 3.857 | 3.8405 | 0.248 | -0.008 | 0.001 |
| 1.4 | 3.911 | 3.857 | 3.8385 | 0.251 | -0.006 | 0.001 |
| 1.45 | 3.9129 | 3.857 | 3.84 | 0.26 | -0.011 | 0.002 |
| 1.5 | 3.9148 | 3.857 | 3.8395 | 0.263 | -0.013 | 0.003 |
| 1.55 | 3.9168 | 3.857 | 3.8383 | 0.269 | -0.011 | 0.003 |

| | | | | | | |
|---|---|---|---|---|---|---|
| 1.6 | 3.9187 | 3.857 | 3.8384 | 0.274 | -0.012 | 0.004 |
| 1.65 | 3.9206 | 3.857 | 3.8376 | 0.279 | -0.012 | 0.004 |
| 1.7 | 3.9226 | 3.857 | 3.8371 | 0.284 | -0.014 | 0.001 |
| 1.75 | 3.9245 | 3.857 | 3.8366 | 0.289 | -0.014 | 0.002 |
| 1.8 | 3.9264 | 3.857 | 3.8354 | 0.294 | -0.013 | 0.002 |
| 1.85 | 3.9284 | 3.857 | 3.8356 | 0.299 | -0.016 | -0.001 |
| 1.9 | 3.9303 | 3.857 | 3.8349 | 0.304 | -0.013 | 0.003 |
| 1.95 | 3.9322 | 3.857 | 3.8342 | 0.309 | -0.012 | 0.003 |
| 2 | 3.9341 | 3.857 | 3.8336 | 0.314 | -0.009 | 0.003 |

**Supplementary Table 2. Landau Free Energy Parameters for $SrTiO_3$**

| Parameters | Value (in cgs units, temperature in K) |
|---|---|
| $\alpha_1$ | $4.5[\coth\left(\frac{54}{T}\right) - \coth\left(\frac{54}{30}\right)] \times 10^{-3}$ |
| $\alpha_{11}$ | $2.5 \times 10^{-12}$ |
| $\alpha_{12}$ | $1.5 \times 10^{-12}$ |
| $c_{11}$ | $3.36 \times 10^{12}$ |
| $c_{12}$ | $1.07 \times 10^{12}$ |
| $c_{44}$ | $1.27 \times 10^{12}$ |
| $g_{11}$ | 1.39 |
| $g_{12}$ | -0.12 |
| $g_{44}$ | 0.27 |
| $\beta_{11}$ | $1.94 \times 10^{43}$ |
| $\beta_{12}$ | $3.96 \times 10^{43}$ |
| $\lambda_{11}$ | $1.3 \times 10^{27}$ |
| $\lambda_{12}$ | $- 2.5 \times 10^{27}$ |
| $\lambda_{44}$ | $- 2.3 \times 10^{27}$ |
| $t_{11}$ | $- 3.74 \times 10^{15}$ |
| $t_{12}$ | $0.15 \times 10^{15}$ |
| $t_{44}$ | $7.0 \times 10^{15}$ |
