# Supplementary material for: Strain-induced room-temperature ferroelectricity in SrTiO3 membranes
Source: Nat Commun. 2020 Jun 19;11:3141. doi: 10.1038/s41467-020-16912-3 (PMC7305178; doi:10.1038/s41467-020-16912-3)
Supplement: Supplementary file 3 — Description of Additional Supplementary Files [file 41467_2020_16912_MOESM3_ESM.pdf]

Description of Additional Supplementary Files

File name: Supplementary Data 1

Description: Crystallographic Information Files which provide the structural details of $SrTiO_3$ lattice in response to the uniaxial strain along the [100] direction
